# SARS Alert Applicability

**DOI:** 10.3201/eid1008.040221

**Published:** 2004-08

**Keywords:** letter, SARS, surveillance, sickness certification

**To the Editor:** Since its emergence early in 2003, the epidemic of severe acute respiratory syndrome (SARS) has been characterized by its rapid spread among healthcare workers. August 14, 2003, the World Health Organization (WHO) issued an alert concerning SARS and recommended a staged approach to surveillance (1). Because occupational transmission has been a feature of the SARS outbreak, WHO recommends surveillance for clusters of alert cases among healthcare workers in low-risk areas (i.e., cases not reported, only imported cases reported, or local cases with limited transmission potential reported). A SARS alert is identified when two or more healthcare workers in the same healthcare unit meet the clinical case definition of SARS with onset of illness in the same 10-day period.

To determine the value of routinely collecting worker absence data as part of this kind of surveillance and to assess a threshold level of possible alert cases, directors of six major Italian hospitals were asked for the number of cases that fit the alert definition in 2003. (In Italy, the hospital director is a physician who is in charge of nosocomial and occupational infection control.) The facilities involved were three general hospitals, two university hospitals, and one research hospital; each has an infectious and respiratory tract diseases unit. Three of four patients with imported cases of probable SARS observed in Italy during the 2003 epidemic (2) were treated in two of these hospitals.

No hospitals were able to immediately provide the requested data; in all hospitals in Italy, information on sickness certificates is recorded only for administrative purposes, and certificates are not generally used for medical surveillance. The European Union Council Directive 89/391 directs all participating countries to introduce measures to improve worker safety and health and to provide a designated service that will protect workers, prevent occupational risks, including hazards from biological agents, and conduct health surveillance. In the hospital, these activities are coordinated by the hospital director. When a worker has a transmissible disease, the attending physician for the infected patient recommends that the patient stay home from work for the duration of the infectivity period. If the illness is included in the list of notifiable infectious diseases, the case must be reported to the local public health authority so infection control measures can be implemented. However, neither the attending physician nor public health personnel usually supervise home isolation, and adherence to the recommendations relies on the patient.

Sickness certificates are generally provided by the physician and sent by the worker to the hospital administration within 3 days of illness onset. The certificate indicates the prognosis (i.e., recommended number of days absent from work) but does not report the diagnosis because of privacy concerns. In case of hospital admission, the worker can send the hospital certificate (attesting to the duration of the hospital stay), followed by a physician's certificate for the recommended length of convalescence, if any.

To determine how the sickness certification system in other European Union countries operates and assesses the feasibility of the WHO alert surveillance, we interviewed specialists in infectious diseases or public health in France (seven imported cases of SARS, two in healthcare workers), Spain (one case), and Denmark (no cases) (2) by electronic mail. According to their answers, the situation in those countries is not substantially different from that in Italy.

In view of the increasing concern related to the emergence and reemergence of transmissible diseases, surveillance efforts focused on groups likely to be first affected by the reemergence of SARS have been strongly encouraged (3,4). Possible alternatives similar to the SARS alert system have been proposed, based on healthcare workers' sickness absenteeism, when other illnesses are concerned. For example, the effectiveness of enforced monitoring of pneumonia in healthcare workers requiring hospitalization should be evaluated in the context of a wider syndromic surveillance strategy (5).

Although the current healthcare worker sickness reporting system cannot be fully representative and generalizable, Italy and several other European Union countries (e.g., France, Spain, and Denmark) do not support initiating the WHO recommendation and do not have the capacity to detect and respond to SARS, should it reemerge. To overcome barriers to early detection of cases and clusters of severe unexplained respiratory infections that might signal the reemergence of SARS, regulatory changes are necessary, and efforts should be made to balance the need for protecting the privacy of persons with the need for an effective surveillance system.

To identify clusters of occupational diseases among healthcare workers and provide prompt response to any alert, an expanded sickness information system should be implemented. For example, an active confidential assessment of diagnosis could be performed in selected circumstances when healthcare workers are absent. We plan to evaluate the feasibility of this kind of surveillance by focusing on workers with absences with longer than a week and on workers with onset of illness in the same 10-day period.
